# *TaCIPK29*, a CBL-Interacting Protein Kinase Gene from Wheat, Confers Salt Stress Tolerance in Transgenic Tobacco

**DOI:** 10.1371/journal.pone.0069881

**Published:** 2013-07-29

**Authors:** Xiaomin Deng, Wei Hu, Shuya Wei, Shiyi Zhou, Fan Zhang, Jiapeng Han, Lihong Chen, Yin Li, Jialu Feng, Bin Fang, Qingchen Luo, Shasha Li, Yunyi Liu, Guangxiao Yang, Guangyuan He

**Affiliations:** The Genetic Engineering International Cooperation Base of Chinese Ministry of Science and Technology, Chinese National Center of Plant Gene Research (Wuhan) HUST Part, Key Laboratory of Molecular Biophysics MoE, College of Life Science and Technology, Huazhong University of Science & Technology (HUST), Wuhan, China; RIKEN Plant Science Center, Japan

## Abstract

Calcineurin B-like protein-interacting protein kinases (CIPKs) have been found to be responsive to abiotic stress. However, their precise functions and the related molecular mechanisms in abiotic stress tolerance are not completely understood, especially in wheat. In the present study, *TaCIPK29* was identified as a new member of *CIPK* gene family in wheat. *TaCIPK29* transcript increased after NaCl, cold, methyl viologen (MV), abscisic acid (ABA) and ethylene treatments. Over-expression of *TaCIPK29* in tobacco resulted in increased salt tolerance, which was demonstrated by higher germination rates, longer root lengths and better growth status of transgenic tobacco plants compared to controls when both were treated with salt stress. Physiological measurements indicated that transgenic tobacco seedlings retained high K^+^/Na^+^ ratios and Ca^2+^ content by up-regulating some transporter genes expression and also possessed lower H_2_O_2_ levels and reduced membrane injury by increasing the expression and activities of catalase (CAT) and peroxidase (POD) under salt stress. Moreover, transgenic lines conferred tolerance to oxidative stress by increasing the activity and expression of CAT. Finally, TaCIPK29 was located throughout cells and it preferentially interacted with TaCBL2, TaCBL3, NtCBL2, NtCBL3 and NtCAT1. Taken together, our results showed that *TaCIPK29* functions as a positive factor under salt stress and is involved in regulating cations and reactive oxygen species (ROS) homeostasis.

## Introduction

Soil salinity, as a severe abiotic stress factor, limits the growth of most plant species and can cause significant losses in crop yield. Salt stress can affect photosynthesis, growth, energy metabolism, lipid metabolism and protein synthesis [1]. However, plants develop a variety of sophisticated mechanisms to protect themselves from salt stress. The major factors of plant responses to salt stress include perception and transduction of stress signals.

Calcium, as a second messenger, plays an important role in various signal transduction pathways [2]. Several classes of calcium-sensing proteins, including calmodulin (CaM), calmodulin-like (CML), calcineurin B-like (CBL) proteins, and calcium-dependent protein kinases (CDPKs), have been identified in plants [3]. Calcineurin B-like protein-interacting protein kinases (CIPKs) belong to a Ca^2+^ mediated CBL-CIPK network in response to stress [4]–[7]. Genome-wide analysis has identified 26 *CIPKs* in Arabidopsis [8], 33 *CIPKs* in rice [6], [8], 27 *CIPKs* in poplar [9] and 43 *CIPKs* in maize [10].

In plants, several CIPKs have been reported to be involved in salt stress responses. Notably, the salt overly sensitive (SOS) pathway is one of the most important signaling pathways in salinity signal transduction [11]–[14]. AtCIPK24/AtSOS2 can interact with AtCBL4/AtSOS3 to function on the Na^+^/H^+^ antiporter, AtSOS1/AtNHX7, enhancing salt stress tolerance in roots [15], whereas the AtCBL10-AtCIPK24 complex protects the shoot tissue from salt stress [16]. AtCIPK24/AtSOS2 has also been found to interact with nucleoside triphosphate kinase 2 (NDPK2) as well as AtCAT2/AtCAT3, which are involved in reactive oxygen species (ROS) signaling and scavenging [17]. This evidence indicated that AtCIPK24/AtSOS2 is a crucial regulator in the salt stress signaling network, which can mediate both Na^+^ homeostasis and the oxidative stress response. Most of the *CIPK* genes enhancing salt tolerance are similar to *AtCIPK24/AtSOS2* such as *MdSOS2, MdCIPK6L* and *ZmCIPK16* [7], [18], [19]. Meanwhile, *AtCIPK6*, *AtCIPK9*, *HbCIPK2*, *SlSOS2* and *AtCIPK23* have been reported to be involved in K^+^ homeostasis, in which *AtCIPK6* and *AtCIPK23* can increase the activity of the plasma membrane K^+^ transporter such as AKT1 [5], [20], [21], [22], [23]. Therefore, further investigations are necessary to understand whether wheat CIPKs can regulate K^+^/Na^+^ homeostasis and ROS scavenging under salt stress. In addition, the interaction between CBLs and CIPKs in wheat is yet to be elucidated.

Internationally, wheat production is affected by many environmental stresses, such as drought, salinity and extreme temperatures. Genetic improvement of stress resistance in wheat is highly desired and understanding the molecular mechanisms of abiotic stress responses is therefore necessary. *CIPK* genes play an important role in regulating abiotic stress tolerance in plants. In comparison to other species, however, little is known about *CIPKs* in wheat. Only one *CIPK* gene, *WPK4,* has been characterized in wheat, which is involved in light, nutrient deprivation and cytokinin signaling [24], [25]. The main function of the CBL-CIPK network in response to abiotic stress has not been characterized in wheat. In this work, we reported a wheat *CIPK* gene *TaCIPK29* that conferred salt tolerance not only by improving the K^+^/Na^+^ ratios and Ca^2+^ content but also by decreasing H_2_O_2_ accumulation and membrane damage.

## Results

### Cloning and Sequence Analysis of the Full-length *TaCIPK29* cDNA

To obtain salt stress responsive *CIPK* genes in wheat, we referred to the highly similar orthologs in rice. Twelve rice *CIPKs* (*OsCIPK7*, *OsCIPK8*, *OsCIPK9*, *OsCIPK10*, *OsCIPK11*, *OsCIPK15*, *OsCIPK16*, *OsCIPK17*, *OsCIPK21*, *OsCIPK22*, *OsCIPK29* and *OsCIPK30*) were reported to be up-regulated by salt stress, whereas only *OsCIPK15* was further studied for salt tolerance in rice [26]. Then, we performed TBLASTN analysis in the DFCI database (http://compbio.dfci.harvard.edu/tgi/) using salt-induced rice CIPK protein sequences and found several wheat tentative consensuses (TCs) of these rice *CIPK* genes (data not shown). Among the wheat CIPK-like TCs, TC371359, which shared 86% similarity and 79% identity with OsCIPK29 in amino acids, encodes a peptide of 306 amino acids that lacked more than 100 amino acid residues in the N-terminal. Importantly, this TC371359 consisted of six expressed sequence tags (ESTs), in which BQ753202 was identified from a salt-stressed root cDNA library (TA065E1X) [27]. These data indicated that TC371359 might be a salt stress responsive *CIPK* gene in wheat.

Employing the rapid amplification of cDNA ends (RACE) technique, we successfully amplified the full-length cDNA designated as *TaCIPK29* (accession no. JX243013). The obtained cDNA of *TaCIPK29* was 1862 bp with an ORF of 1311 bp. The TaCIPK29 protein consists of 436 amino acid residues with a predicted relative molecular mass of 47.4 kDa. Multiple sequence alignment suggested that the deduced TaCIPK29 protein contained an N-terminal highly conserved kinase domain, the FISL domain and the PPI domain located in the C-terminal [28] (Fig. S1). Phylogenetic analysis of TaCIPK29 with other CIPKs from Arabidopsis, tobacco, rice and barley indicated that TaCIPK29 was close to OsCIPK29, HvCIPK29, AtCIPK14 and NtCIPK14 in the intron-less subgroup (Fig. S2). To confirm whether *TaCIPK29* contained an intron in the wheat genome, we cloned *TaCIPK29* by using cDNA and the genomic DNA of wheat as template. The resulted sequences showed *TaCIPK29* harbored no intron (data not shown). These results indicated that *TaCIPK29* obtained in this study is a member of the *CIPK* family in wheat.

### *TaCIPK29* is Expressed in Different Wheat Tissues

**Figure 1:**
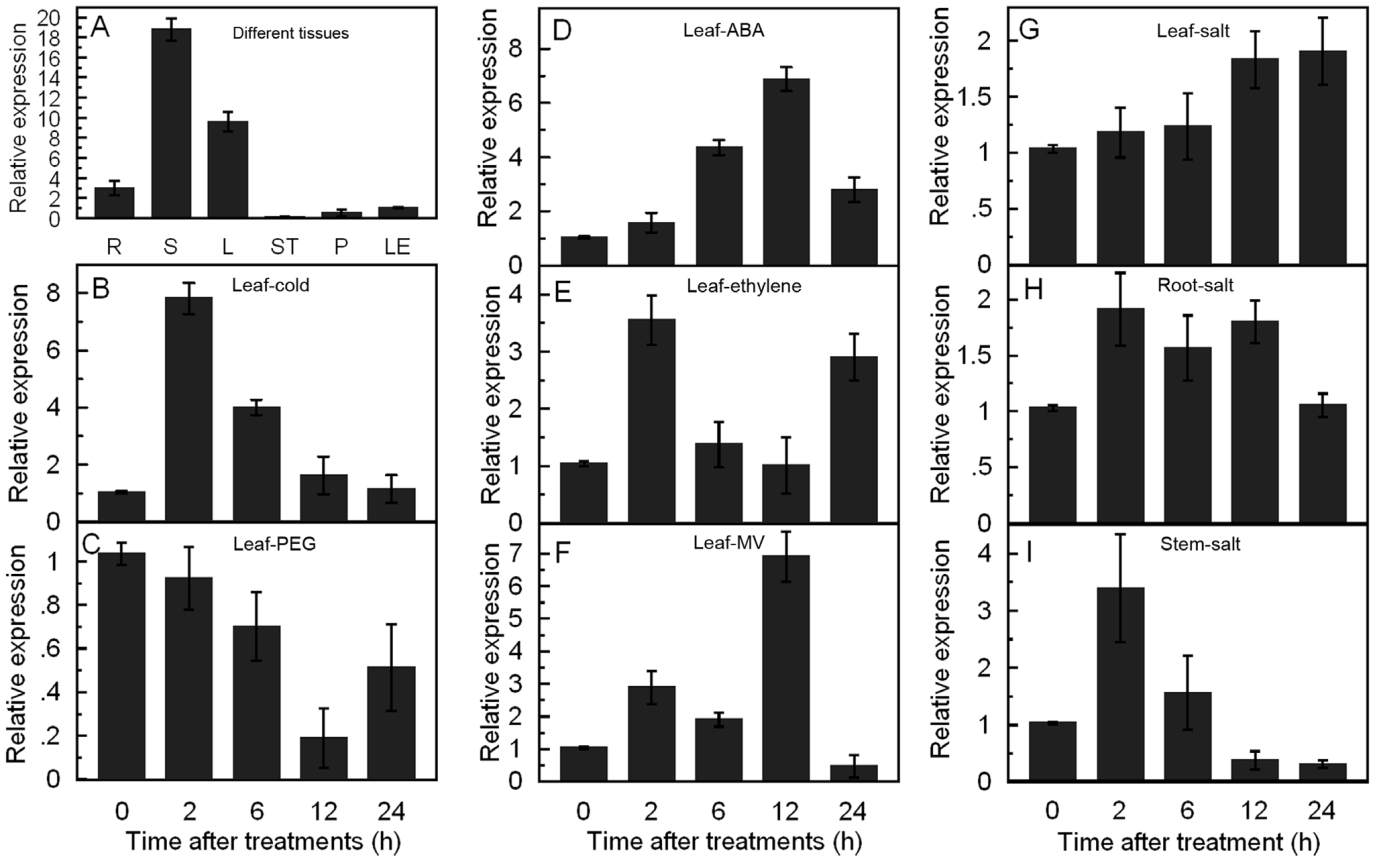
Expression patterns of *TaCIPK29* in different tissues in wheat and changes in expression after treatments with PEG6000, NaCl, MV, ABA, and ethylene by qRT-PCR analysis. A: different organs (R: root; S: stem; L: leaf; ST: stamen; P: pistil; LE: lemma); B: low temperature treatment; C: 20% PEG treatment; D: 100 µM ABA treatment; E: 100 µM ethylene treatment; F: 30 µM MV treatment; G–I: 200 mM NaCl treatment. B–G: leaves samples; H: roots samples; I: stems samples. Vertical bars indicate ±SE of four replicates on one sample. Three biological experiments were performed with similar results.

To assess the expression of *TaCIPK29* in various wheat tissues, mRNAs were isolated from different tissues of wheat including root, stem, leaf, stamen, pistil and lemma. Quantitative real-time polymerase chain reaction (qRT-PCR) analysis revealed that *TaCIPK29* was expressed in all the above tissues, of which the stem and leaf had higher expression levels compared to the other tissues examined (Fig. 1A).

### *TaCIPK29* Transcript is Responsive to Abiotic Stress, ABA and Ethylene

To determine the response of *TaCIPK29* to abiotic stress, wheat leaves were sampled to determine *TaCIPK29* transcript levels after PEG6000, NaCl, low temperature and MV treatment. The results suggested that the transcript of *TaCIPK29* was strongly up-regulated by low temperature and MV treatment, marginally induced by NaCl treatment and inhibited by PEG6000 treatment in leaves (Figs. 1B, 1C, 1F and 1G). To further confirm the upregulation of *TaCIPK29* under NaCl treatment, qRT-PCR was performed with wheat roots and stems as samples. The results suggested that the expression of *TaCIPK29* was obviously induced in stems and marginally induced in roots under NaCl treatment (Figs. 1H and 1I). Various signal molecules play important roles in response to abiotic stress, especially for ABA and ethylene. Thus, the effects of ABA and ethylene on *TaCIPK29* transcription were also examined, which indicated that the *TaCIPK29* transcript increased after ABA and ethylene treatments (Figs. 1D and 1E).

### Generation of Transgenic Tobacco Plants Overexpressing *TaCIPK29*

**Figure 2:**
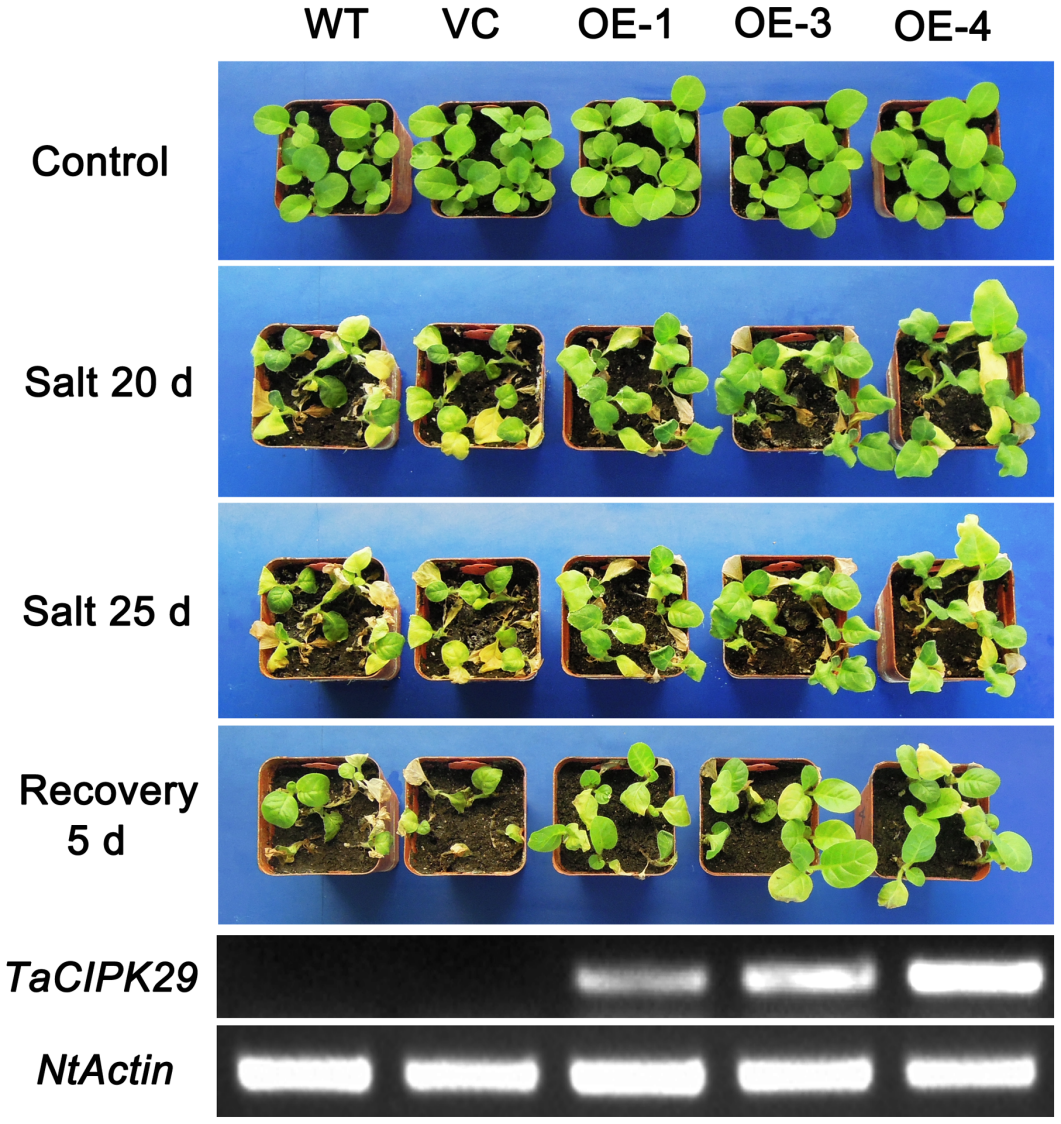
The analysis of salt tolerance in transgenic lines and controls (VC and WT). The phenotype of the four-week-old seedlings of transgenic tobacco plants and controls (VC and WT) grown in pots were recorded when plants grown in the absence (control) or in the presence of 300 mM NaCl for 20 and 25 d as well as plants subjected to a 5 d recovery after a treatment with 300 mM NaCl for 25 d. The whole two-week-old seedlings of transgenic plants and control plants (VC and WT) were used to extract RNA to detect *TaCIPK29* expression by RT-PCR with *NtActin* as an internal control. Three biological experiments were carried out with similar results.

To further detect the function of *TaCIPK29* in abiotic stress tolerance, transgenic tobacco plants over-expressing *TaCIPK29* under the control of the *Cauliﬂower Mosaic Virus* (CaMV) 35S promoter were generated. A total of 18 transgenic lines (T_1_) were identified by kanamycin-resistant and PCR analysis using primers specific to *TaCIPK29* and the green fluorescence protein (GFP) gene (data not shown). Among the T_1_ lines, three (OE-1, OE-3 and OE-4) exhibited a ratios of ∼3∶1 segregation on the basis of kanamycin resistance. Moreover, seedlings from all three transgenic T_2_ lines grew well on Murashige and Skoog (MS) medium with 150 mg/L of kanamycin. In addition, tobacco plants transformed with empty vector alone were also subjected to similar analysis. Expression of *TaCIPK29* was also determined by RT-PCR, which indicated that *TaCIPK29* mRNA was present in all three transgenic lines but not in the vector control (VC) lines and wild-type (WT). Among the T_2_ lines, OE-3 and OE-4 had higher *TaCIPK29* expression levels (Fig. 2).

### Over-expression of *TaCIPK29* Enhances Salt Tolerance in Transgenic Tobacco

**Figure 3:**
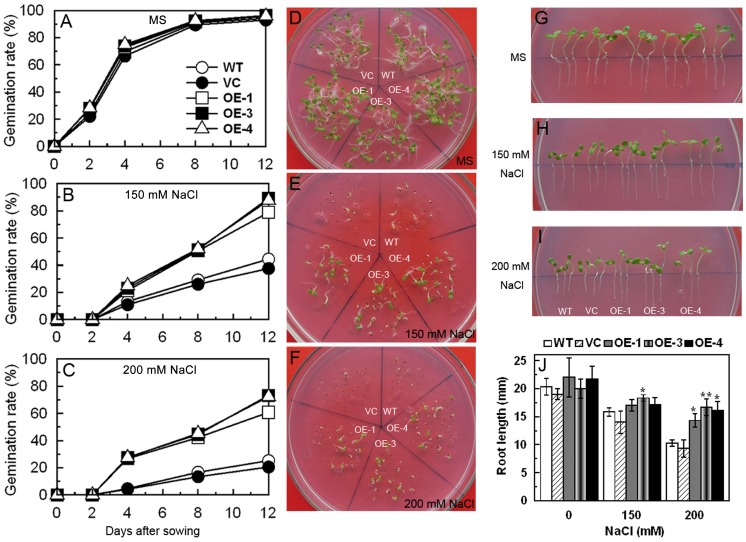
The analysis of salt tolerance in *TaCIPK29*-overexpressing plants and controls (VC and WT) at early developmental stage. A total of 200 surface-sterilized seeds of each transgenic line, WT or VC germinated for 12 days on MS medium containing 0 (A, D), 150 (B, E) or 200 mM (C, F) NaCl. Panels A, B and C show the seed germination rates. Panels D, E and F show photographs of plants after 12 days of germination. One-week-old tobacco seedlings were transplanted to MS or MS supplied with 150 or 200 mM NaCl for one week. Panels G, H and I show photographs of seedlings treated or not with NaCl Panel J shows root length measurements Vertical bars indicate ±SD calculated from four replicates (with 3 seedlings per replicate). Three biological experiments were done with similar results.

Four-week-old tobacco seedlings grown in pots were treated with 300 mM NaCl to characterize the performance of *TaCIPK29* transgenic lines under salt stress in soil. After 20 days or 25 days NaCl treatment, *TaCIPK29* transgenic plants survived better with less leaf wilting compared to WT and VC tobacco plants (Fig. 2). Moreover, after recovery, the transgenic lines exhibited stronger survival than WT and VC plants (Fig. 2). In a second experiment, seeds from all three transgenic lines and control lines (WT and VC) were germinated on MS or MS with 150 mM or 200 mM NaCl for 12 d. *TaCIPK29*-overexpressing lines showed a significantly higher germination rate on the MS medium containing 150 mM and 200 mM NaCl although little difference was observed between controls and transgenic lines under normal conditions (Figs. 3A–F). In addition, tobacco seedlings from transgenic lines and the controls (VC and WT) were cultured on MS for one week, and then were transplanted to MS or MS with 150 mM or 200 mM NaCl for one week to examine the root length. Results showed that transgenic lines displayed lesser suppression of root elongation than the controls (VC and WT) under 150 mM or 200 mM NaCl treatment (Figs. 3G–J). There were no significant differences between controls and the transgenic lines grown on MS. These data suggested that tobacco plants overexpressing *TaCIPK29* enhanced salt stress tolerance.

### Over-Expression of *TaCIPK29* Maintains high K^+^/Na^+^ Ratios and Ca^2+^ Content Under Salt Stress

**Figure 4:**
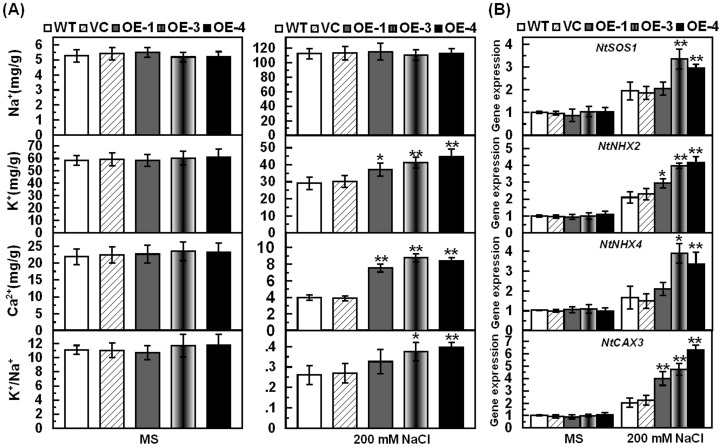
Ion content and expression analysis of transporter genes in transgenic lines and controls (VC and WT) under normal and salt stress conditions. Two-week-old seedlings were transplanted to MS or MS with 200 mM NaCl for one week. Whole seedlings were sampled to measure the content of Na^+^, K^+^, Ca^2+^ and the ratio of K^+^/Na^+^ was calculated (A). Two-week-old tobacco seedlings were transplanted to MS or MS with 200 mM NaCl for two days and the whole seedlings were used to measure the expression of transporter genes (B). Data are means ±SD calculated from four replicates. Asterisks indicate significant difference between the WT and the three transgenic lines (*p<0.05; **p<0.01). Three different experiments were performed with similar results.

To detect the accumulation of Na^+^, K^+^ and Ca^2+^ in the controls (WT and VC) and transgenic lines, the concentration of these cations was measured in tobacco plants under normal and salt stress conditions. Under normal conditions, there were no significant differences in the content of Na^+^, K^+^ and Ca^2+^ nor in the K^+^/Na^+^ ratios in the whole seedlings of the controls (WT and VC) and transgenic lines (Fig. 4A). However, under salt stress conditions, transgenic lines had elevated K^+^ and Ca^2+^ content compared to the two controls although no difference was detected between the transgenic lines and the two controls in Na^+^ contents (Fig. 4A). Further analysis indicated that the transgenic lines retained higher K^+^/Na^+^ ratios than the controls under salt stress (Fig. 4A). These results suggested that over-expression of *TaCIPK29* had an effect on regulating the contents of these cations, which were involved in maintaining higher K^+^/Na^+^ ratios and Ca^2+^ content in transgenic tobacco plants under salt stress.

### Over-expression of *TaCIPK29* Elevates the Expression of *NtSOS1*, *NtNHX2*, *NtNHX4* and *NtCAX3*

AtSOS1, which is activated by AtSOS2/AtCIPK24, is responsible for Na^+^ export from the cell and enhancing salt stress tolerance [29]. Additionally, both LeNHX2 and LeNHX4 function in K^+^ compartmentalization and Na^+^ and K^+^ transport from the cytosol to cell compartments and are induced in *SlSOS2*/*SlCIPK24*-overexpressing transgenic tomato under salt stress [23], [30]. The Arabidopsis vacuolar H^+^/Ca^2+^ antiporter CAX3 is required for Ca^2+^ homeostasis and salt stress tolerance under NaCl treatment [31], [32]. To identify the tobacco orthologs of AtSOS1, LeNHX2, LeNHX4 and AtCAX3, BLASTP, TBLASTN and phylogenetic analysis were carried out. The results showed that NtSOS1, NtNHX2, NtNHX4 and NtCAX3 shared high similarity and identity with AtSOS1, LeNHX2, LeNHX4 and AtCAX3, respectively (Table S2). Additionally, on the evolutionary timescale, NtSOS1, NtNHX2, NtNHX4 and NtCAX3 were very close to AtSOS1, LeNHX2, LeNHX4 and AtCAX3 respectively (Figs. S4 and S5). These results suggested that NtSOS1, NtNHX2, NtNHX4 and NtCAX3 from tobacco are true orthologs of AtSOS1, LeNHX2, LeNHX4 and AtCAX3, respectively. To gain a deeper understanding of *TaCIPK29* function in salt stress tolerance, transcript levels of these transporter genes were detected in the controls (VC and WT) and the transgenic lines under normal and salt stress conditions. The results showed that the expression levels of *NtSOS1, NtNHX2, NtNHX4* and *NtCAX3* were higher in transgenic lines than that in controls under salt conditions although there was no significant difference between transgenic lines and the two controls under normal conditions (Fig. 4B).

### Over-expression of *TaCIPK29* Improves Antioxidant Enzyme Activities and Reduces the Content of H_2_O_2_ and Malonaldehyde (MDA) and Ion Leakage (IL) Under Salt Stress

**Figure 5:**
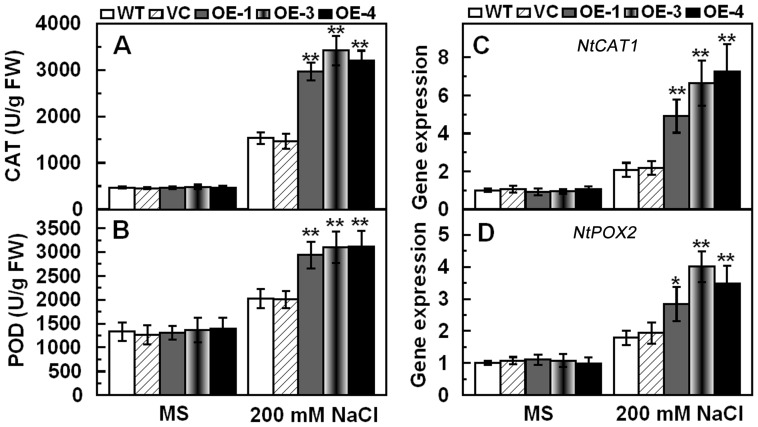
Activities and expression of CAT and POD in the controls (WT and VC) and transgenic plants grown under normal and salt stress conditions. Two-week-old tobacco seedlings were transplanted to MS or MS with 200 mM NaCl for one week. Whole seedlings were sampled to measure activities of CAT and POD (A, B). Two-week-old tobacco seedlings were transplanted to MS or MS with 200 mM NaCl for two days and the whole seedlings were used to examine the expression of *NtCAT1* and *NtPOX2* (C, D). Data are means ±SD calculated from four replicates. Asterisks indicate significant difference between the WT and the three transgenic lines (*p<0.05; **p<0.01). Three different experiments were performed with similar results.

**Figure 6:**
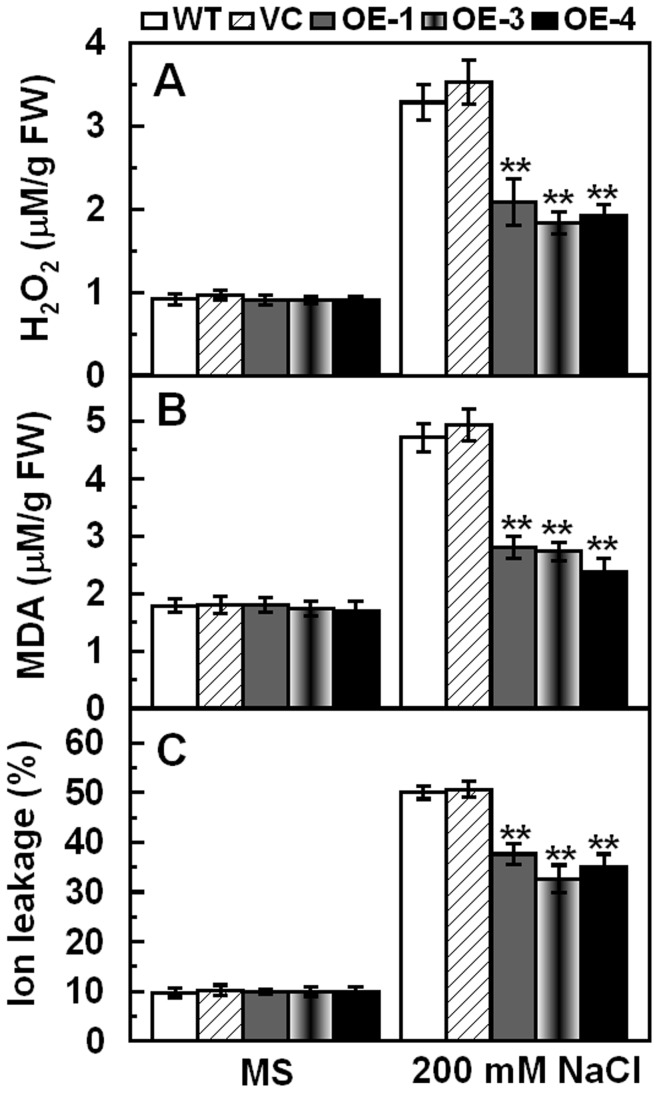
Analysis of H_2_O_2_, MDA and IL in the controls (WT and VC) and transgenic lines under normal and salt stress conditions. Two-week-old tobacco seedlings were transplanted to MS or MS supplied with 200 mM NaCl for one week. Whole seedlings were sampled to measure H_2_O_2_ (A), MDA (B) and IL (C). Data are means ±SD calculated from four replicates. Asterisks indicate significant difference between the WT and the three transgenic lines (*p<0.05; **p<0.01). Three biological experiments were done with similar results.

Because Na^+^, K^+^ and Ca^2+^ were involved in cell metabolism, enzyme activation and protein biosynthesis, activities of antioxidantive enzymes such as catalase (CAT; EC 1.11.1.6) and peroxidase (POD; EC 1.11.1.7) were detected under normal conditions and salt treatment. After 7 days salt treatment, the three transgenic lines maintained higher activities of POD and CAT than WT and VC (Figs. 5A and 5B). Accordingly, *NtCAT1* and *NtPOX2* transcripts were also higher in transgenic lines than in WT and VC under salt conditions (Figs. 5C and 5D). The activity and expression of SOD was similar in the controls (VC and WT) and transgenic lines under normal and salt stress conditions (data not shown). Antioxidative enzymes play crucial roles in ROS scavenging and thereby relieve membrane lipid peroxidation. Therefore, IL and the contents of H_2_O_2_ and MDA were further examined, which indicated that transgenic lines showed lower H_2_O_2_ accumulation, IL and MDA contents than the controls (VC and WT) under salt stress conditions (Figs. 6A, 6B and 6C). These results suggested that over-expression of *TaCIPK29* improved expression and activities of POD and CAT, and reduced H_2_O_2_ accumulation and membrane damage under salt stress.

### Over-expression of *TaCIPK29* Enhances Oxidative Stress Tolerance in Transgenic Tobacco

**Figure 7:**
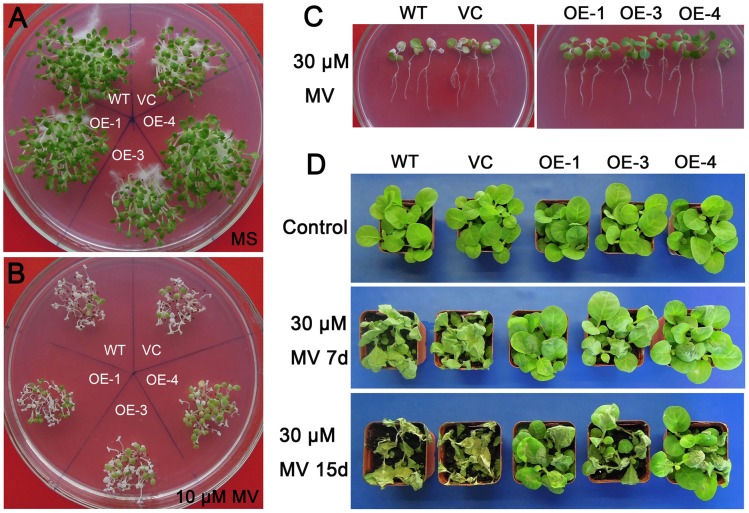
Analysis of the enhanced oxidative stress tolerance in transgenic lines. Tobacco seeds germinated on MS for two weeks (A). Tobacco seeds germinated on MS with 10 µM MV for two weeks (B). Two-week-old tobacco plants were transferred to MS supplied with 30 µM MV for one week (C). Five-week-old tobacco plants were subjected to 30 µM MV treatment for 7 days or 15 days (D). Three biological experiments were conducted with similar results.

**Figure 8:**
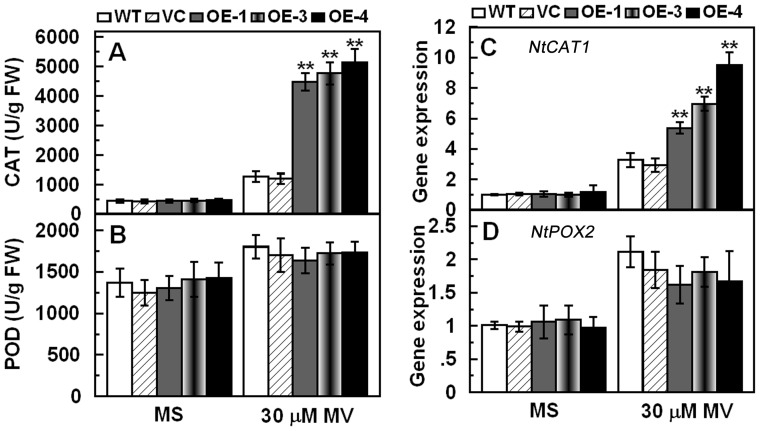
Activities and expression of CAT and POD in the controls (WT and VC) and transgenic lines grown under normal and oxidative stress conditions. Two-week-old tobacco seedlings were transplanted to MS or MS supplied with 30 µM MV for one week. Whole seedlings were sampled to detect activities of CAT and POD (A, B). Two-week-old tobacco seedlings were transplanted to MS or MS supplied with 30 µM MV for two days and the whole seedlings were used to measure the transcript levels of *NtCAT1* and *NtPOX2* (C, D). Data are means ±SD calculated from four replicates. Asterisks indicate significant difference between the WT and the three transgenic lines (*p<0.05; **p<0.01). Three different experiments were performed with similar results.

**Figure 9:**
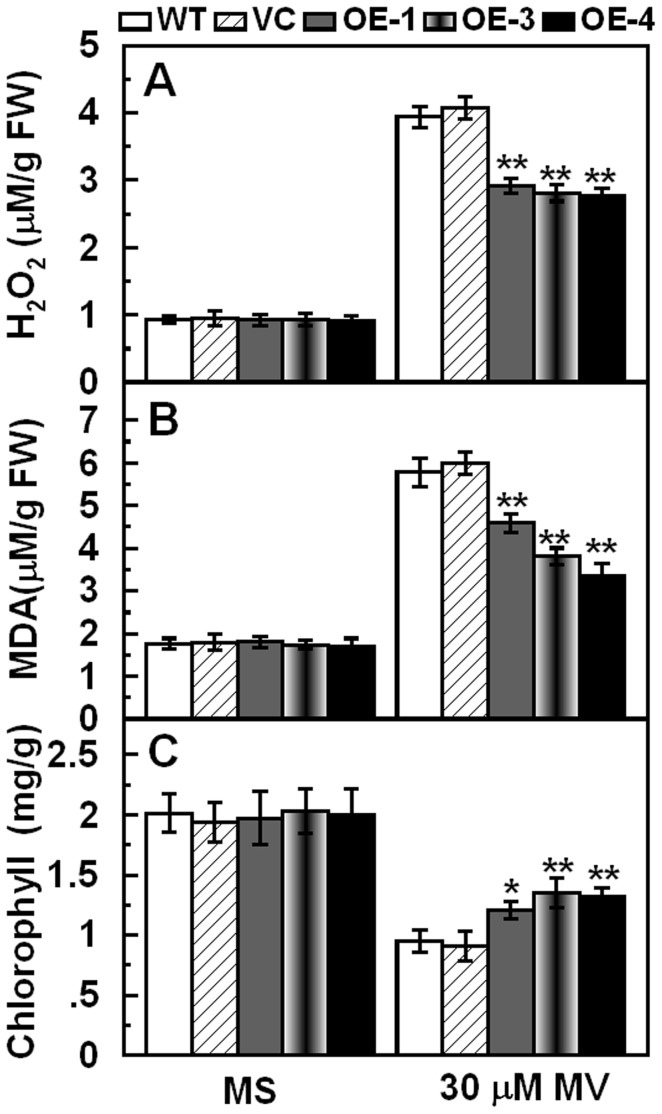
Analysis of H_2_O_2_, MDA and chlorophyll contents in the controls (WT and VC) and transgenic lines grown under normal and oxidative stress conditions. Two-week-old tobacco seedlings were transplanted to MS or MS supplied with 30 µM MV for one week. Whole seedlings were sampled to measure H_2_O_2_ (A), MDA (B) and chlorophyll (C). Data are means ±SD calculated from four replicates. Asterisks indicate significant difference between the WT and the three transgenic lines (*p<0.05; **p<0.01). Three different experiments were performed with similar results.

To further assess the role of *TaCIPK29* in regulating antioxidant mechanisms, the correlation between *TaCIPK29* and oxidative stress was explored. Tobacco seeds were germinated on MS or MS with 10 µM MV for two weeks, which showed that chlorosis was more severe in WT and VC than in transgenic lines (Figs. 7A and 7B). In the second experiment, two-week-old tobacco seedlings were transferred to MS with 30 µM methyl viologen for one week. The cotyledon bleaching was also more severe in WT and VC than in transgenic lines (Fig. 7C). Moreover, after 7 days or 15 days MV treatment, transgenic lines exhibited more tolerance to oxidative stress than WT and VC in soil (Fig. 7D). Analysis of activities and expression of antioxidant enzymes indicated that two controls (VC and WT) exhibited lower CAT activities and expression than the transgenic lines under MV treatment (Fig. 8). Furthermore, physiological investigation indicated that transgenic tobacco plants contained higher chlorophyll and lower H_2_O_2_ and MDA contents than the two controls (VC and WT) under MV treatment (Fig. 9). These results suggested that over-expression of *TaCIPK29* reduced H_2_O_2_ accumulation by enhancing the antioxidant system under oxidative stress.

### TaCIPK29 Interacts with TaCBL2, TaCBL3, NtCBL2, NtCBL3 and NtCAT1 in Yeast Two-hybrid Assays

**Figure 10:**
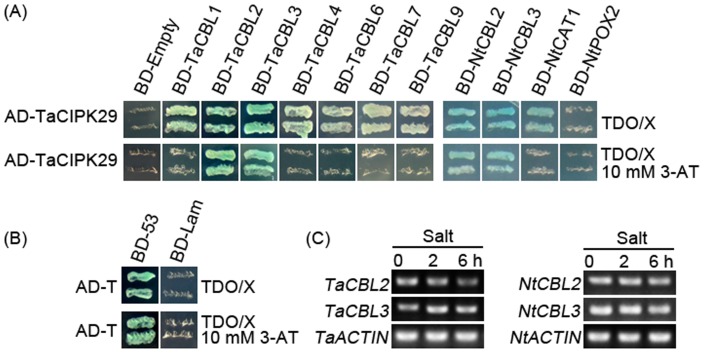
Interaction between TaCIPK29 and CBLs/CAT and expression analysis of *CBLs*. TaCIPK29 was fused to the GAL4 activation domain (AD) and TaCBLs/NtCBLs/NtCAT1/NtPOX2 were fused to the GAL4 DNA-binding domain (BD). The interactions between TaCIPK29 and TaCBLs/NtCBLs/NtCAT1/NtPOX2 were examined in the triple dropout medium containing 20 µg/ml X-α-gal (SD/−His/−Leu/−Trp plus X-α-gal, TDOX) or TDOX adding 10 mM 3-AT (A). The interaction between SV40 large T-antigen (AD-T) and murine p53 (BD-53) used as positive control and the interaction between SV40 large T-antigen and human lamin C (BD-Lam) used as negative control were tested on the same TDOX medium and TDOX medium containing 10 mM 3-AT (B). Two-week-old wheat and tobacco seedlings were treated with 200 mM NaCl to detect the expression of CBLs (C). Three different experiments were performed with similar results.

To further understand the regulating mechanism of TaCIPK29, yeast two-hybrid analysis was performed. TaCIPK29 was fused to the activation domain vector (pGADT7) as prey, whereas the TaCBLs were fused to the binding domain vector (pGBKT7) as bait. The yeast cells co-transformed with the AD-TaCIPK29 and distinct BD vectors were spared in the selective medium TDO/X (Figs. 10A and 10B). The results showed that TaCIPK29 could interact with TaCBL1, TaCBL2, TaCBL3 and TaCBL4, but very weakly with TaCBL6 and TaCBL7. In addition, 3-ami-notriazole (3-AT), as an inhibitor of His synthetase, was often used to identify the strong interaction through inhibiting the weak interaction of the pray and the bait [33]. When we supplied the selective medium with 10 mM 3-AT and 20 µM X-α-gal, we found that only the yeast cell with the AD-TaCIPK29 and BD-TaCBL2 or AD-TaCIPK29 and BD-TaCBL3 grew and exhibited blue color. The results suggested that TaCIPK29 had strong interactions with TaCBL2 and TaCBL3. Furthermore, NtCBL2 and NtCBL3 shared high similarity with TaCBL2 and TaCBL3 (Fig. S3) were also cloned and introduced into the BD vector, and showed their capability to interact with TaCIPK29 in the yeast two-hybrid assays (Fig. 10). Because the expression of *NtPOX2* and *NtCAT1* was higher in *TaCIPK29*-overexpressing tobacco plants than that in the controls (WT and VC) under salt stress, NtPOX2 and NtCAT1 were also introduced into BD vector to detect their interaction with TaCIPK29. The results indicated that there were no interactions between TaCIPK29 and NtPOX2, whereas TaCIPK29 could interact with NtCAT1 (sharing high similarity with AtCAT2) (Fig. 10). The interactions between TaCIPK29 and NtSOS1/NtNHX2/NtNHX4/NtCAX3 were not conducted because of the lack of complete sequences for the four tobacco genes. These results suggested TaCIPK29 interacted with NtCAT1 and CBL2/CBL3 preferentially.

### Both *TaCIPK29* and *TaCBL3* Expressions are Induced by Salt Treatment

RT-PCR was applied to investigate the expression of *TaCIPK29-CBL2/CBL3* components under salt stress (Fig. 10C). The results showed that *TaCBL3* were induced by NaCl treatment, while the expression of *TaCBL2* was inhibited by NaCl treatment. In addition, *NtCBL2* and *NtCBL3* displayed constant expression levels under NaCl treatment. These results implied that TaCBL3-TaCIPK29 components may be involved in salt stress response in wheat.

### TaCIPK29 is Located in the Nucleus, Cytoplasm, and Plasma Membrane

**Figure 11:**
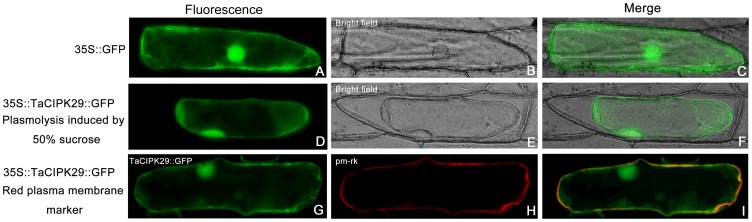
Subcellular localization analysis of TaCIPK29-GFP fusions in onion epidermal cells. (A) p35S::GFP fluorescence; (B) bright-field image of p35S::GFP; (C) overlapped image of p35S::GFP; (D) p35S::TaCIPK29-GFP fluorescence; (E) bright-field image of p35S::TaCIPK29-GFP; (F) overlapped image of p35S::TaCIPK29-GFP; (G) p35S::TaCIPK29-GFP fluorescence; (H) pm-rk (plasma membrane-localized maker) red fluorescence; (I) merged image of (G) and (H). (D-F) are images of onion epidermal cells after plasmolysis induced by 50% sucrose solution.

Subcellular localization of the TaCIPK29 protein was investigated in a transient expression assay with 35S::TaCIPK29-GFP (PBI121- *TaCIPK29*-*GFP*) translational fusion in onion epidermal cells using particle bombardment. The results showed that the fluorescence of cells transformed with TaCIPK29-GFP was distributed throughout the cell, including the nucleus, cytoplasm and plasma membrane, when these cells were in normal conditions (Fig. 11G) or after plasmolysis (Figs. 11D, 11E and 11F). Furthermore, to confirm the plasma membrane localization of TaCIPK29, both plasma membrane marker (PM-RK) and TaCIPK29-GFP fusion protein were expressed in the onion epidermis cells [34]. The red PM-RK and green fluorescence were both observed in the plasma membrane (Figs. 11G, 11H and 11I). In addition, ﬂuorescent signals were found throughout cells when expressing GFP alone (Figs. 11A, 11B and 11C). These results indicated that the TaCIPK29-GFP fusion protein located to the nucleus, plasma membrane and cytoplasm.

## Discussion

As is already known, Na^+^ has an adverse effect on cell metabolism and its uptake and distribution strongly affect the salt sensitivity of plants [1]. However, plants have developed some biochemical and molecular mechanisms to cope with the negative effects of salinity. Well-characterized genes include Na^+^/H^+^ antiporter *SOS1*, the Na^+^ inﬂux transporter family *HKT* and the tonoplast Na^+^/H^+^ antiporter family *NHX* [1], [35]. In addition, a large number of *CIPKs* have been characterized to play important roles in salt stress tolerance, such as *AtSOS2*, *AtCIPK6*, *AtCIPK9*, *AtCIPK23*, *AtCIPK24*, *OsCIPK15* and *SlCIPK24* etc. However, these studies mainly focused on the model plants Arabidopsis, rice and tomato. The precise role of *CIPKs* in salt stress tolerance has not been completely understood, especially for crops. Although more than 35 *CIPK* genes have been identified to date in the wheat genome, only the function of *WPK4* was reported. In this study, a *CIPK* gene, designated as *TaCIPK29*, was cloned and characterized from wheat.

### *TaCIPK29* Plays a Positive Role Under Salt Stress

A number of studies have demonstrated the function of *CIPKs* in salt stress tolerance. The expression of *TaCIPK29* was induced after treatment with NaCl, implying that it may be involved in the response to salt stress (Fig. 1). Therefore, to further assess the function of *TaCIPK29* under salt stress, transgenic tobacco plants overexpressing *TaCIPK29* were generated. As indicated in Figures 2 and 3, one week old and four weeks old tobacco plants overexpressing *TaCIPK29* had enhanced tolerance to salt stress relative to WT and VC. These results were in line with some previous studies on *CIPK* genes improving salt stress tolerance in transgenic plants.

### Improved Tolerance to Salt Stress is Associated with Higher K^+^/Na^+^ Ratios and Ca^2+^ content through regulating transporter genes

The functions of *CIPKs* on modulating Na^+^ and K^+^ homeostasis under salt stress have been characterized in previous studies. AtCIPK24/AtSOS2 can interact with AtCBL4/AtSOS3 to function on the Na^+^/H^+^ antiporter, AtSOS1/AtNHX7, enhancing salt stress tolerance in roots [15]. Meanwhile, *AtCIPK6*, *AtCIPK9*, *HbCIPK2*, *SlSOS2* and *AtCIPK23* have been reported to function in K^+^ homeostasis, in which *AtCIPK6* and *AtCIPK23* can increase the activity of the plasma membrane K^+^ transporter such as AKT1 [5], [20], [21], [22], [23]. In addition, Ca^2+^, which is crucial for plants to combat abiotic stress, mediates the CBL-CIPK network in response to stress [4]–[7]. Thus, in the present study, the content of these cations in the whole seedlings of the controls (WT and VC) and transgenic lines were measured under normal conditions and salt stress. The results showed that over-expression of *TaCIPK29* elevated K^+^/Na^+^ ratios and Ca^2+^ content under salt stress (Fig. 4A). *NtSOS1*, *NtNHX2*, *NtNHX4* and *NtCAX3* are orthologs of *AtSOS1*, *LeNHX2*, *LeNHX4* and *AtCAX3* respectively as shown in Figures S4 and S5 and Table S2. Moreover, the transgenic plants exhibited higher expression of *NtSOS1*, *NtNHX2*, *NtNHX4* and *NtCAX3* compared to the WT and VC under salt stress (Fig. 4B). AtSOS1 and SlSOS1 are membrane-bound Na^+^/H^+^ antiporters that function in the enhancement of salt stress tolerance by exporting Na^+^ [15], [29]. However, the endosomal K^+^, Na^+^/H^+^ antiporter LeNHX2 and the vacuolar Na^+^, K^+^/H^+^ antiporter LeNHX4 were related to K^+^ compartmentalization and to Na^+^ and K^+^ transport from the cytosol to cell compartments [23], [30]. Therefore, it is concluded that the up-regulation of *NtSOS1* is beneficial for exporting Na^+^, while the increased expression of *NtNHX2* and *NtNHX4* leads to Na^+^ compartmentalization, avoiding its toxicity in the cytosol. As a result, the transgenic lines contained similar total Na^+^ content to the two controls under salt conditions. In addition, the upregulation of *NtNHX2* and *NtNHX4* in transgenic lines compared to WT and VC may contribute to K^+^ absorption for transgenic plants. The Arabidopsis vacuolar H^+^/Ca^2+^ antiporter CAX3 is required for Ca^2+^ homeostasis and salt stress tolerance under NaCl treatment [31], [32]. The expression of *NtCAX3* was upregulated in *TaCIPK29*-overexpressing plants compared to the two controls, implying that it was associated with elevated Ca^2+^ content in transgenic plants. Thus, the improved tolerance to salt stress was related to higher K^+^/Na^+^ ratios and Ca^2+^ content through regulating transporter genes in transgenic tobacco plants.

### The Antioxidative System is Involved in *TaCIPK29* Improving Tolerance to Salt Stress

Plant photosynthesis is often affected by excess Na^+^ *via* generating excess ROS, which destroys cell membrane. However, plant cells can accumulate K^+^ to combat the harmful influence of excess Na^+^ under salt stress because K^+^ is necessary for protection of cellular enzymes including the antioxidant enzymes [36], [37]. Therefore, higher K^+^/Na^+^ ratios maintained in plants under salt stress contribute to the cellular ROS homeostasis. Moreover, Ca^2+^ is also involved in activating the antioxidative defense system to reduce the H_2_O_2_ levels in plants [38]–[42]. Antioxidative enzymes were also reported to have positive effect on salt stress tolerance in plants [1], [43]. In this study, analysis of the antioxidant enzymes indicated that the transgenic lines had higher expression levels and enzyme activities of POD and/or CAT than the two controls under salt and MV treatment (Figs. 5 and 8). Moreover, TaCIPK29 could also interact with NtCAT1 directly (Fig. 10). These results suggested that over-expression of *TaCIPK29* improved the activation of the antioxidant defense system, which was benefit for preventing transgenic lines from ROS-mediated injury under salt or oxidative stress. AtCIPK24/AtSOS2 was also reported to interact with NDPK2 as well as with AtCAT2 and AtCAT3, which are involved in ROS signaling and scavenging [17]. Most of the *CIPK* genes enhancing salt tolerance are similar to *SOS2* such as *MdSOS2, MdCIPK6L* and *ZmCIPK16* [7], [18], [19]. Here, we found that there was an interaction between TaCIPK29 and NtCAT1 (Fig. 10), indicating that the increased expression and activities of CAT may be due to the TaCIPK29 function on NtCAT1 directly.

### TaCBL3-TaCIPK29 Components may be Involved in Salt Stress Tolerance

CBLs, a kind of Ca^2+^ sensor, can perceive and decode the Ca^2+^ signature generated under specific stress. CIPK complexes were activated by CBLs to regulate the downstream effectors [44]. An interaction between TaCIPK29 and TaCBLs was detected, which indicated that TaCIPK29 had a strong interaction with TaCBL2 and TaCBL3 (Fig. 10). Further, tobacco CBLs, NtCBL2 and NtCBL3 were confirmed to have a strong interaction with TaCIPK29. Therefore, the function of TaCIPK29 is mainly regulated by CBL2/3 in wheat and tobacco. Moreover, both *TaCBL3* and *TaCIPK29* expression were upregulated by NaCl, implying that TaCBL3-TaCIPK29 components may be involved in response to salt stress in wheat. AtCBL2 and AtCBL3 were confirmed to strongly interact with AtCIPK14 (homologs of TaCIPK29) [45]. However, the roles of these interactions in salt stress signaling transduction are still unclear. Although *OsCBL3* and *OsCIPK29* were strongly induced by salt [4], [46], the interaction between OsCBL3 and OsCIPK29 and their function has not yet been identified. Here, we established that TaCBL3-TaCIPK29 components may be involved in salt stress tolerance.

In conclusion, the findings of this study demonstrated that *TaCIPK29* plays a positive role in salt stress tolerance. *TaCIPK29* conferred tolerance to salt stress not only by increasing K^+^/Na^+^ ratios and Ca^2+^ content through up-regulating some transporter genes but also by decreasing H_2_O_2_ accumulation and membrane injury through enhancing the expression and activities of antioxidant enzymes.

## Materials and Methods

### Plant Materials and Treatments

The sterilized wheat seeds were germinated in an incubator (25^o^C, 200 µM m^−2^s^−1^, 16 h light/8 h dark cycle). The two-week-old seedlings were transferred to an incubator at 4^o^C for cold stress treatment and sampled at 0 h, 2 h, 6 h, 12 h and 24 h after treatment. PEG and salt treatments were applied by irrigating the two-week-old seedlings with 20% PEG6000 or 200 mM NaCl, respectively, and sampled at 0 h, 2 h, 6 h, 12 h and 24 h after treatment. Plant hormones and oxidative stress treatments were conducted by spraying the seedlings with different chemicals (including 100 µM ABA, 100 µM ethylene and 30 µM MV) and were sampled at 0 h, 2 h, 6 h, 12 hand 24 h after treatment. To detect the expression profile in different tissues, various tissues of wheat (including roots, stems, leaves, pistils, stamens and lemma) were harvested. The samples were stored at −70°C for further analysis.

### Gene Cloning and Sequence Analysis

The wheat ESTs were acquired by searching DFCI and NCBI databases. In order to identify putative salt-responsive *CIPK* genes in wheat, a BLASTN search was performed with the amino acid sequences of 12 salt related OsCIPKs (OsCIPK7, OsCIPK8, OsCIPK9, OsCIPK10, OsCIPK11, OsCIPK15, OsCIPK16, OsCIPK17, OsCIPK21, OsCIPK22, OsCIPK29 and OsCIPK30) to identify the tentative consensus sequences (TCs) of the putative *CIPKs*. Among the TCs, TC371359 showed high identity of amino acid sequence with OsCIPK29 and contained an EST sequence (BQ753202) isolated from a salt-stressed wheat root cDNA library. Thus, TC371359 was seen as a candidate in response to salt stress and was chosen for further functional investigation. Sequence analysis showed that the 5′- and 3′- ends were missing. Then RACE technique was conducted to isolate the 5′- and 3′- ends of *TaCIPK29* using the 5′ RACE primers (P1 and P2, Table S1) and 3′ RACE primers (P3 and P4, Table S1). The cDNA templates for the RACE reaction were acquired from leaves of wheat seedlings that were treated with 20% PEG6000, 200 mM NaCl and cold (4°C) for 2 h. The full-length cDNA sequence of *TaCIPK29* was amplified by PCR with the primers (P5, Table S1). The 5′end of *NtCBL2* was missing and isolated by 5′RACE reaction based on the sequence of the EST (EB440776) with the 5′ RACE primer (P13, Table S1). The full length sequence of *NtCBL2* gene was also obtained by PCR with the specific primers (P14, Table S1). The *NtCBL3*, *NtCAT1* and *NtPOX2* genes were isolated from tobacco by PCR reaction with the primers (P17, P19, and P20, Table S1). Amino acid sequence alignments and the construction of a phylogenetic tree were performed using the Clustalx 2.0 and MEGA 4.0 software, respectively.

### Identification of Tobacco Orthologs of AtSOS1, LeNHX2, LeNHX4 and AtCAX3

TBLASTN was performed in Sol Genomisc Network database (http://solgenomics.net/) and NCBI database (http://blast.ncbi.nlm.nih.gov/Blast.cgi) on the basis of the amino acid sequences of AtSOS1, LeNHX2, LeNHX4 and AtCAX3. Four tobacco orthologs that shared the highest similarity with AtSOS1, LeNHX2, LeNHX4 and AtCAX3 were identified and designated as NtSOS1 (SGN-E793961), NtNHX2 (FG179556), NtNHX4 (EB438297) and NtCAX3 (TC135005) respectively. Tobacco amino acid sequences NtSOS1, NtNHX2, NtNHX4 and NtCAX3 were further confirmed by BLASTP or TBLASTN alignment in NCBI database and phylogenetic analysis with other NHXs or CAXs from Arabidopsis and tomato.

### Gene Expression Assay

Total RNA extraction was performed by using a RNAprep pure plant total RNA extraction kit (TIANGEN, Beijing, China). The RNA was then transcribed into cDNA using a RevertAid™ First Strand cDNA Synthesis kit (Fermentas, Canada). Expression of *TaCIPK29* in different wheat tissues with or without treatments and stress-associated genes in tobacco were analyzed by qRT-PCR. The primers (P6-P7, P21-P27, Table S1) used in qRT-PCR were designed after excluding the highly conserved protein domains. These primers had high specificity and efficiency, which were determined by agarose gel electrophoresis and melting curve analysis using the Opticon monitor 2 qRT-PCR software. PCR products amplified by the primer pairs were sequenced to confirm specificity. The qRT-PCR assay was conducted with three replicates using the SYBR Green PCR master mix kit (ToYoBo, Japan). The relative level of gene expression was detected using the 2^–ΔΔCt^ method (Livak and Schmittgen 2001) [47]. In this method, ΔΔCt = (C_T, Target_ - C_T, Actin_) _Time x_ - (C_T, Target_ - C_T, Actin_) _Time 0_. The C_T_ (cycle threshold) values for both the target and internal control genes were the means of the triplicate PCRs. For the tissue specific assay, the expression of *TaCIPK29* in lemma was regarded as standard. For the expression analysis in wheat after various treatments, untreated samples were determined as calibrators. For gene expression in transgenic tobacco, WT samples under normal conditions were used as calibrators. Amplification efficiencies for the primer pairs were between 0.92 and 1.17. The semi RT-PCR reaction was used to assess the expression of *TaCBL2* (P11, Table S1) and *TaCBL3* (P12, Table S1) as well as *NtCBL2* (P15, Table S1) and *NtCBL3* (P16, Table S1) under salt stress. *TaActin* (P7, Table S1) or *NtACTIN* (P27, Table S1) were used as the internal control for wheat and tobacco, respectively.

### Yeast Two-hybrid Assays

A Matchmaker Gold Yeast Two-Hybrid System (Clontech) was used to examine the interaction between TaCIPK29 and CBLs/NtCAT/NtPOD *in vivo*. The coding sequences of *TaCIPK29* and different candidate genes were amplified by PCR with specific primers (P8, P16, P17, P19, P20, Table S1) harboring restriction sites were cloned into the yeast two-hybrid pGADT7 vector (prey) and the pGBKT7 vector (bait), respectively. The yeast strain Y2HGold was co-transformed with pAD-TaCIPK29 and each pBD (TaCBL1, TaCBL2, TaCBL3, TaCBL4, TaCBL6, TaCBL7 and TaCBL9 as well as NtCBL2, NtCBL3, NtCAT1 and NtPOX2) with the lithium acetate method according to the protocol (Clontech). The co-transformants were screened on triple dropout medium (TDO/X medium, SD/−His/−Leu/−Trp/X-α-gal) without 3-ami-notriazole (3-AT) and with 10 mM 3-AT, respectively. All the yeast two-hybrid studies were performed in triplicate.

### Subcellular Localization Assay

The recombinant plasmid pBI121*-TaCIPK29-GFP* was constructed containing the coding sequence of *TaCIPK29* (P9, Table S1) with a *Xba*I/*Bam*HI restriction site and was used for a sublocalization assay. The pBI121*-TaCIPK29-GFP* and pBI121-*GFP* were transferred into onion epidermal cells by using gene gun bombardment (PDS-1000, BIO-RAD). Fluorescence was examined by a microscope (OLYMPUS IX71, Japan) after 24 h incubation. The fluorescence in onion epidermal cells after plasmolysis induced by 50% sucrose was also recorded. To confirm the localization of TaCIPK29 in the plasma membrane (PM), the PM marker (PM-RK) was applied.

### Generation of Transgenic Tobacco Plants

The plasmid pBI121-*TaCIPK29-GFP* was transformed into tobacco plants using *Agrobacterium tumefaciens* strain LBA4404 according to the transformation method [48]. Positive transgenic plants overexpressing *TaCIPK29* were first screened on MS containing 150 mg/L of kanamycin and then identified by PCR with the primers for *TaCIPK29* (P10, Table S1). The expression of *TaCIPK29* in the 3 independent T_2_ transgenic lines was detected by using RT-PCR with primers (P10 for *TaCIPK29* and P27 for *NtACTIN*, Table S1).

### Stress Tolerance Analysis of the VC, WT and Transgenic Lines

Tobacco seeds (about 200) were surface-sterilized and germinated on MS medium containing different concentrations of NaCl (0 mM, 150 mM or 200 mM) in the incubator (25^o^C) for 12 d and then the germination rate was assessed. For root length assessment, the two controls and transgenic lines grown on MS without NaCl for one week were transferred to MS or MS with 150 mM and 200 mM NaCl for one week in the vertical position. For phenotype assay under salt stress, the sterilized seeds were sown in MS medium and grown for one week and then transferred to the soil container for another three weeks of growth. Then, the four-week-old tobacco seedlings were exposed to 300 mM NaCl for 20 d and 25 d salt stress, and the plants were subsequently recovered for 5 d. For oxidative stress assay, the sterilized seeds germinated on MS medium with 10 µM MV for two weeks. The sterilized seeds grew on MS medium for two weeks and then the seedlings were transferred to MS plates containing 30 µM MV for one week. Five-week-old plants in soil were exposed to 30 µM MV solution for 15 d and the phenotype was recorded.

### Physiological Indices Measurement

The activities of SOD and CAT were spectrophotometrically measured by using Detection Kit (A001 and A007, Jiancheng, Nanjing, China) following the protocols. POD activity measurement was performed according to Polle et al. (1994) [49]. H_2_O_2_ content was determined as described in a previous study [50]. MDA content was measured based on the method described by Heath and Packer (1968) [51]. Chlorophyll was measured by using UV spectrophotometry in accordance with the protocol of Yang et al. (2009) [52]. IL was detected according to the method as described by Jiang and Zhang (2001) with slight modification using a conductivity meter (DDBJ-350, Shanghai, China) instrument. The whole seedlings were firstly washed with distilled water for three times and then incubated in 30 ml distilled water at 25^o^C for 12 h followed by a measurement of the initial conductivity (C1). Then, the samples were boiled for 30 min. After cooling down to the room temperature, the measurement of the final conductivity (C2) was then performed. The relative ion leakage (IL) was calculated using the formula: IL (%) = C1/C2×100. Ion content measurements followed the methods described by Li et al. [5]. Two-week-old tobacco seedlings were transplanted on MS or MS with 200 mM NaCl for one week. The whole seedlings were dried at 80°C for 3 d, and then about 50 mg samples were digested with 6 mL concentrated HNO_3_ overnight. The samples were mixed with 2 mL 30% H_2_O_2_ and heated at 180°C for 15 min. Then the digested samples were diluted to 50 mL and analyzed by inductively coupled plasma-mass spectrometry (ICP-MS).

### Statistical Analysis

Statistical analyses were conducted using Microsoft Excel and SPSS (Chicago, IL, USA). An analysis of variance was employed to compare statistical differences on the basis of Student’s *t* test.

## Supporting Information

Figure S1 **Amino acid sequence alignment of TaCIPK29 with closer homologs from rice and barley.** Black shadings indicate identical residues, while grey shadings represent similar residues. The activation loop, FISL domain and PPI domain are underlined. Sequences of OsCIPK29 (Q7XIW5) and HvCIPK29 (BAJ95612) were acquired from Uniprot database.
(TIF)

Figure S2 **Phylogenetic analysis of TaCIPK29 with other known CIPKs from Arabidopsis, tobacco, rice and barely.** The phylogenetic tree is generated with amino acid sequences by using MEGA4.0 software. The distinct subgroups of CIPK are depicted with the vertical line. The CIPKs sharing high homology with TaCIPK29 are depicted on gray background. The accession numbers of 26 AtCIPKs from *Arabidopsis thaliana* in NCBI database (http://www.ncbi.nlm.nih.gov/) are as follows: AtCIPK06 (AAF8650), AtCIPK07 (AAK16682), AtCIPK08 (AAK16683), AtCIPK09 (AAK16684), AtCIPK10(AAK16685), AtCIPK11(AAK16686), AtCIPK12 (AAK16687), AtCIPK13 (AAK16688), AtCIPK14 (AAK16689), AtCIPK15 (AAK16692), AtCIPK16 (AAF19215), AtCIPK17 (AAK64513), AtCIPK18 (AAK59695), AtCIPK19 (AAK50347), AtCIPK20 (AAK61493), AtCIPK21 (AAK59696), AtCIPK22 (AAL47845), AtCIPK23 (AAK61494), AtCIPK24 (AAK72257), AtCIPK25 (AAL41008), AtCIPK26 (NP_850861 ). Other sequences used: OsCIPK29 (Q7XIW5), HvCIPK29 (BAJ95612.1), NtCIPK14 (KC429561).
(TIF)

Figure S3 **Amino acid alignment and domain analysis of wheat TaCBL2 and TaCBL3 with tobacco NtCBL2 and NtCBL3.** All the CBLs have four calcium binding EF-hand and a FPSF motif. The FPSF motif and the EF-hand (E-helix, F-helix and calcium binding loop) are underlined. The asterisk represents the highly conserved Ser residue (S) that can be phosphorylated in the FPSF motif.
(TIF)

Figure S4 **Phylogenetic analysis of tobacco NHXs with NHXs from Arabidopsis and tomato.** The phylogenetic tree was generated using ClustalX2.0 and MEGA4.0 softwares. Their accession numbers in uniprot database (http://www.uniprot.org/) are as follows: AtNHX1(Q68KI4);AtNHX2(Q56XP4);AtNHX3(Q84WG1);AtNHX4(Q8S397);AtNHX5(Q8S396);AtNHX6(Q8RWU6);AtNHX7/AtSOS1(Q9LKW9);LeNHX1(Q93YH2);LeNHX2(Q93YH1);LeNHX3(Q1JRA3);LeNHX4(Q1JRA2); SlSOS1(Q4W3B5).
(TIF)

Figure S5 **Phylogenetic analysis of NtCAX3 with CAXs from Arabidopsis and tomato.** The phylogenetic tree was generated using ClustalX2.0 and MEGA4.0 softwares. AtCAXs are obtained from uniprot database (http://www.uniprot.org/). SlCAXs are obtained from plantGDB database (http://www.plantgdb.org/SlGDB/or ftp://ftp.plantgdb.org/download/Genomes/SlGDB/). Their accession numbers are as follows:AtCAX1(Q39253);AtCAX2(Q39254);AtCAX3(Q93Z81);AtCAX4(Q945S5);AtCAX5(Q8L783);AtCAX6(Q9LFZ8);AtCAX7(Q9FKP1);AtCAX8(Q9FKP2);AtCAX9(Q9LJI2);AtCAX10(Q9SYG9);AtCAX11(O04034);Solyc01g098800;Solyc02g069710; Solyc03g032240; Solyc03g123790; Solyc06g006110; Solyc06g009130; Solyc07g006370; Solyc07g042000; Solyc07g056110; Solyc07g062700; Solyc09g005260 (SlCAX3); Solyc09g072690; Solyc12g011070; Solyc12g014110; Solyc12g055750.
(TIF)

Table S1 **Primers used for PCR analysis.**
(DOC)

Table S2 **Sequence analysis of tobacco antiporters with the corresponding orthologs in Arabidopsis and tomato.**
(DOC)
