# Supplementary figures and images for: TaCIPK29, a CBL-Interacting Protein Kinase Gene from Wheat, Confers Salt Stress Tolerance in Transgenic Tobacco
Source: PLoS One. 2013 Jul 29;8(7):e69881. doi: 10.1371/journal.pone.0069881 (PMC3726728; doi:10.1371/journal.pone.0069881)

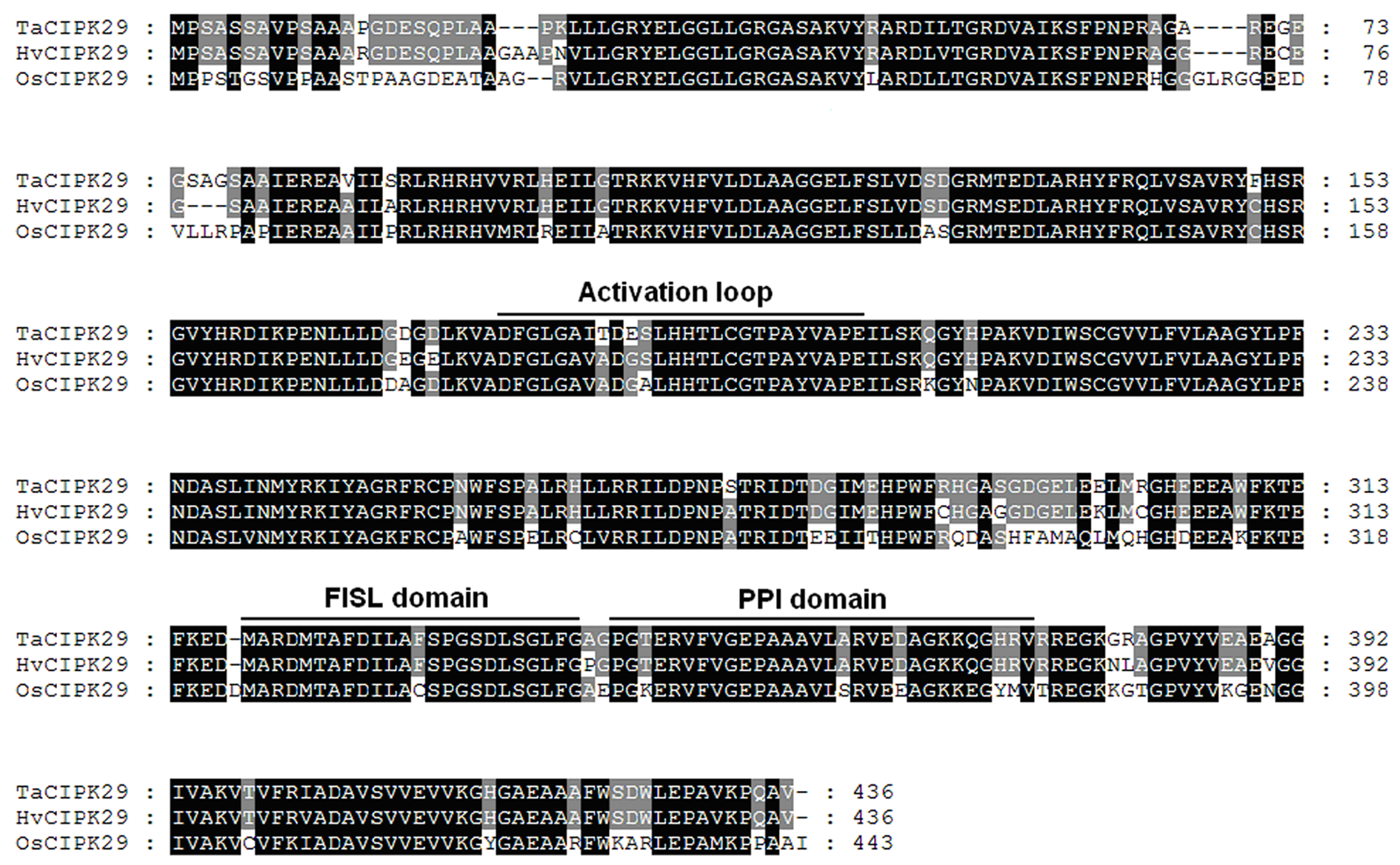

Supplement: Figure S1 — Amino acid sequence alignment of TaCIPK29 with closer homologs from rice and barley. Black shadings indicate identical residues, while grey shadings represent similar residues. The activation loop, FISL domain and PPI domain are underlined. Sequences of OsCIPK29 (Q7XIW5) and HvCIPK29 (BAJ95612) were acquired from Uniprot database. (TIF) [file pone.0069881.s001.tif]

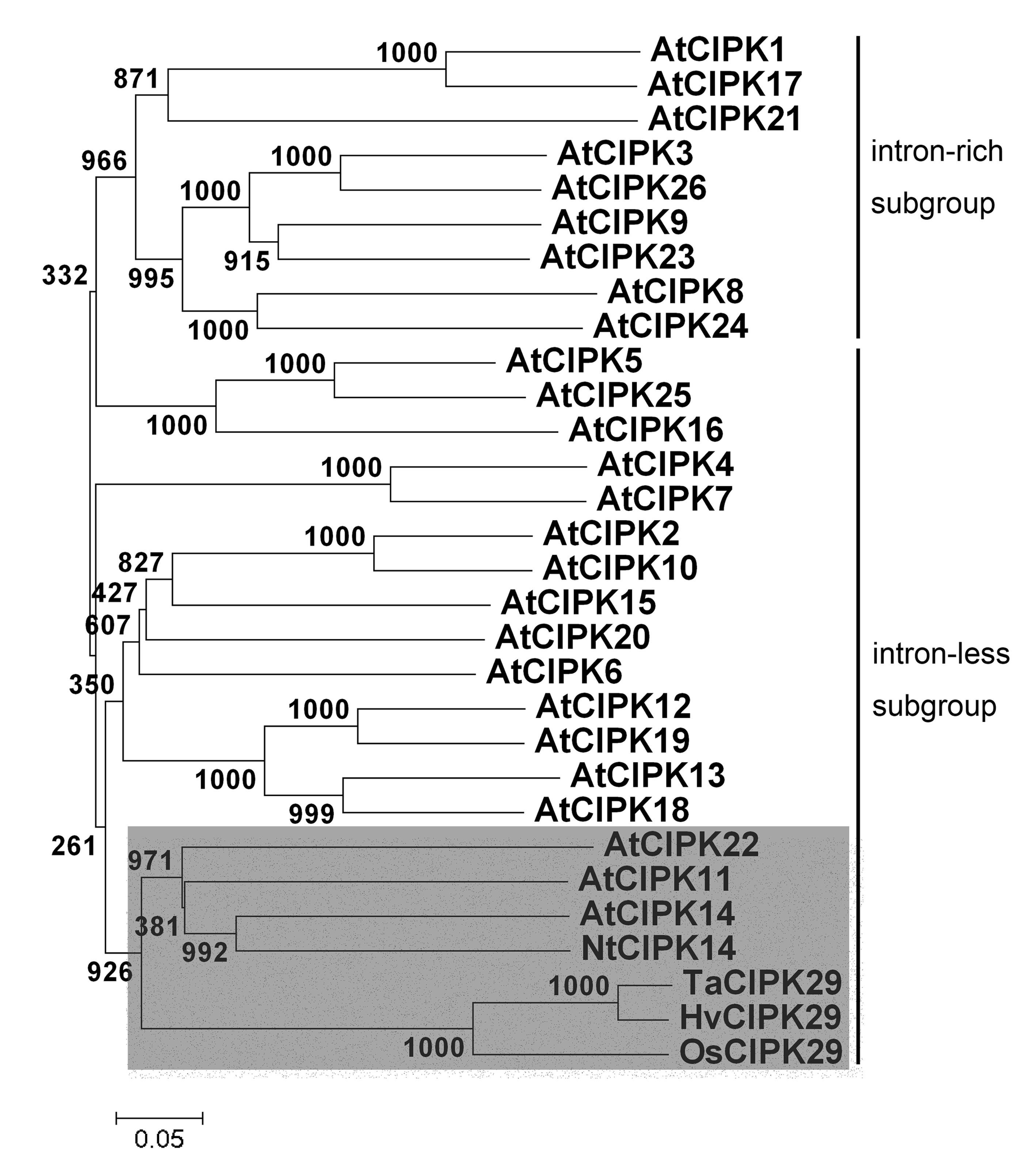

Supplement: Figure S2 — Phylogenetic analysis of TaCIPK29 with other known CIPKs from Arabidopsis, tobacco, rice and barely. The phylogenetic tree is generated with amino acid sequences by using MEGA4.0 software. The distinct subgroups of CIPK are depicted with the vertical line. The CIPKs sharing high homology with TaCIPK29 are depicted on gray background. The accession numbers of 26 AtCIPKs from Arabidopsis thaliana in NCBI database (http://www.ncbi.nlm.nih.gov/) are as follows: AtCIPK06 (AAF8650), AtCIPK07 (AAK16682), AtCIPK08 (AAK16683), AtCIPK09 (AAK16684), AtCIPK10(AAK16685), AtCIPK11(AAK16686), AtCIPK12 (AAK16687), AtCIPK13 (AAK16688), AtCIPK14 (AAK16689), AtCIPK15 (AAK16692), AtCIPK16 (AAF19215), AtCIPK17 (AAK64513), AtCIPK18 (AAK59695), AtCIPK19 (AAK50347), AtCIPK20 (AAK61493), AtCIPK21 (AAK59696), AtCIPK22 (AAL47845), AtCIPK23 (AAK61494), AtCIPK24 (AAK72257), AtCIPK25 (AAL41008), AtCIPK26 (NP_850861 ). Other sequences used: OsCIPK29 (Q7XIW5), HvCIPK29 (BAJ95612.1), NtCIPK14 (KC429561). (TIF) [file pone.0069881.s002.tif]

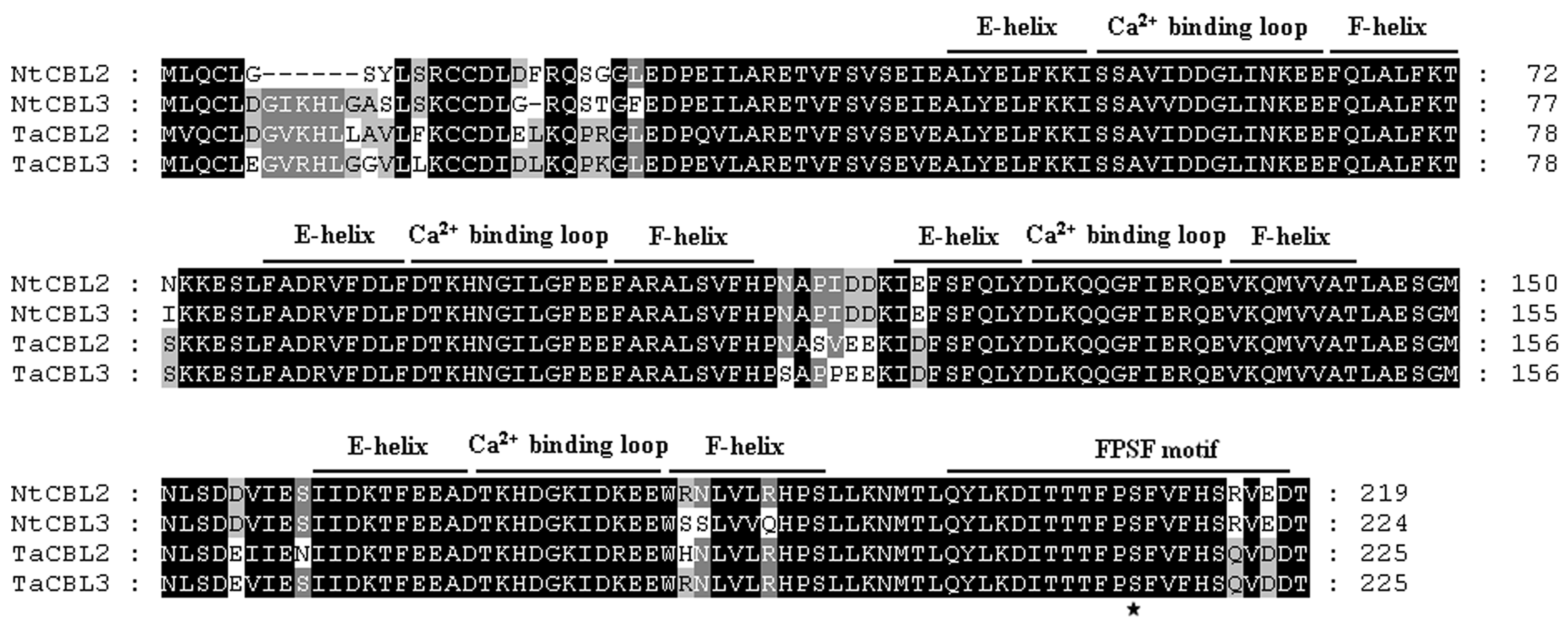

Supplement: Figure S3 — Amino acid alignment and domain analysis of wheat TaCBL2 and TaCBL3 with tobacco NtCBL2 and NtCBL3. All the CBLs have four calcium binding EF-hand and a FPSF motif. The FPSF motif and the EF-hand (E-helix, F-helix and calcium binding loop) are underlined. The asterisk represents the highly conserved Ser residue (S) that can be phosphorylated in the FPSF motif. (TIF) [file pone.0069881.s003.tif]

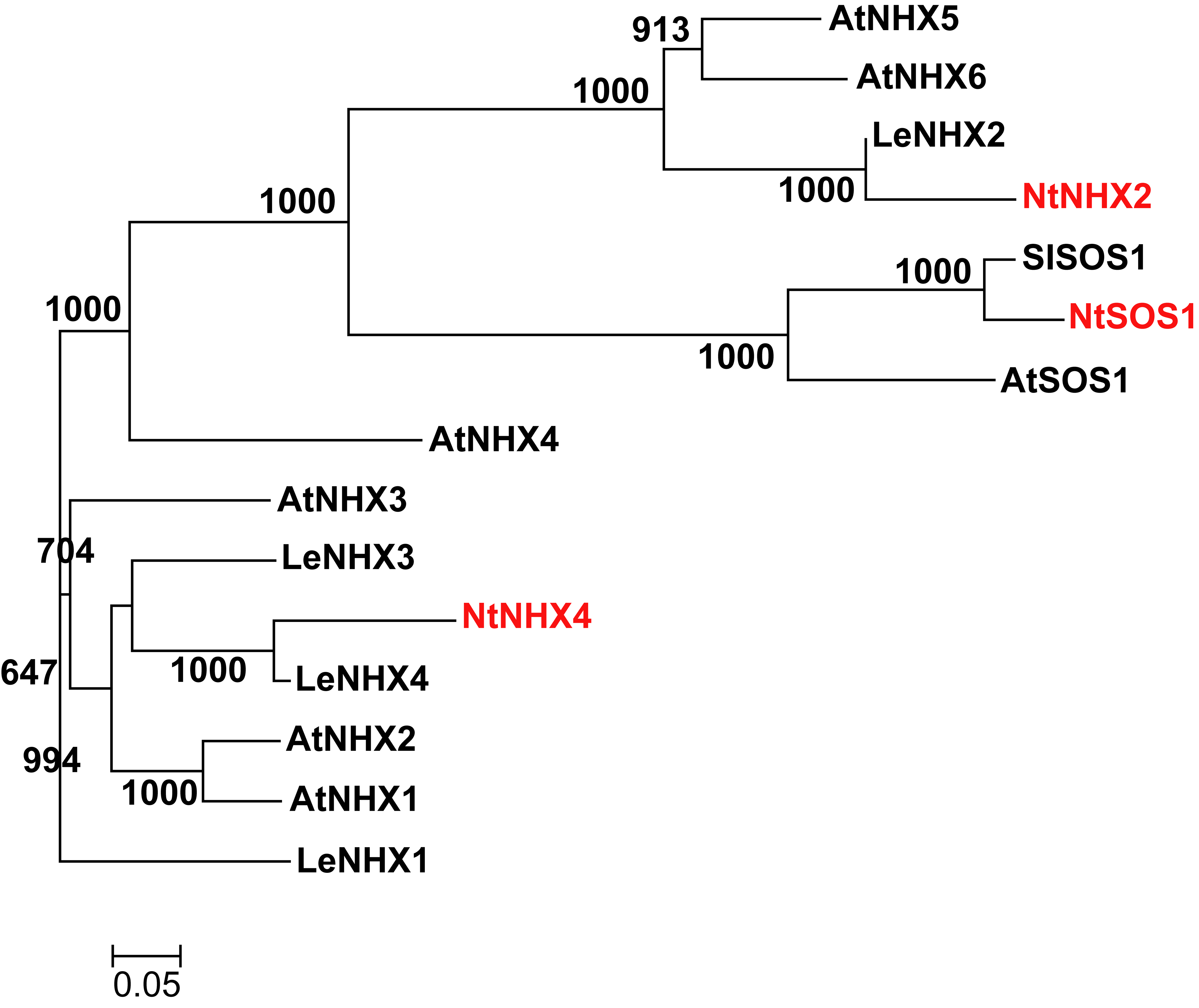

Supplement: Figure S4 — Phylogenetic analysis of tobacco NHXs with NHXs from Arabidopsis and tomato. The phylogenetic tree was generated using ClustalX2.0 and MEGA4.0 softwares. Their accession numbers in uniprot database (http://www.uniprot.org/) are as follows: AtNHX1(Q68KI4);AtNHX2(Q56XP4);AtNHX3(Q84WG1);AtNHX4(Q8S397);AtNHX5(Q8S396);AtNHX6(Q8RWU6);AtNHX7/AtSOS1(Q9LKW9);LeNHX1(Q93YH2);LeNHX2(Q93YH1);LeNHX3(Q1JRA3);LeNHX4(Q1JRA2); SlSOS1(Q4W3B5). (TIF) [file pone.0069881.s004.tif]

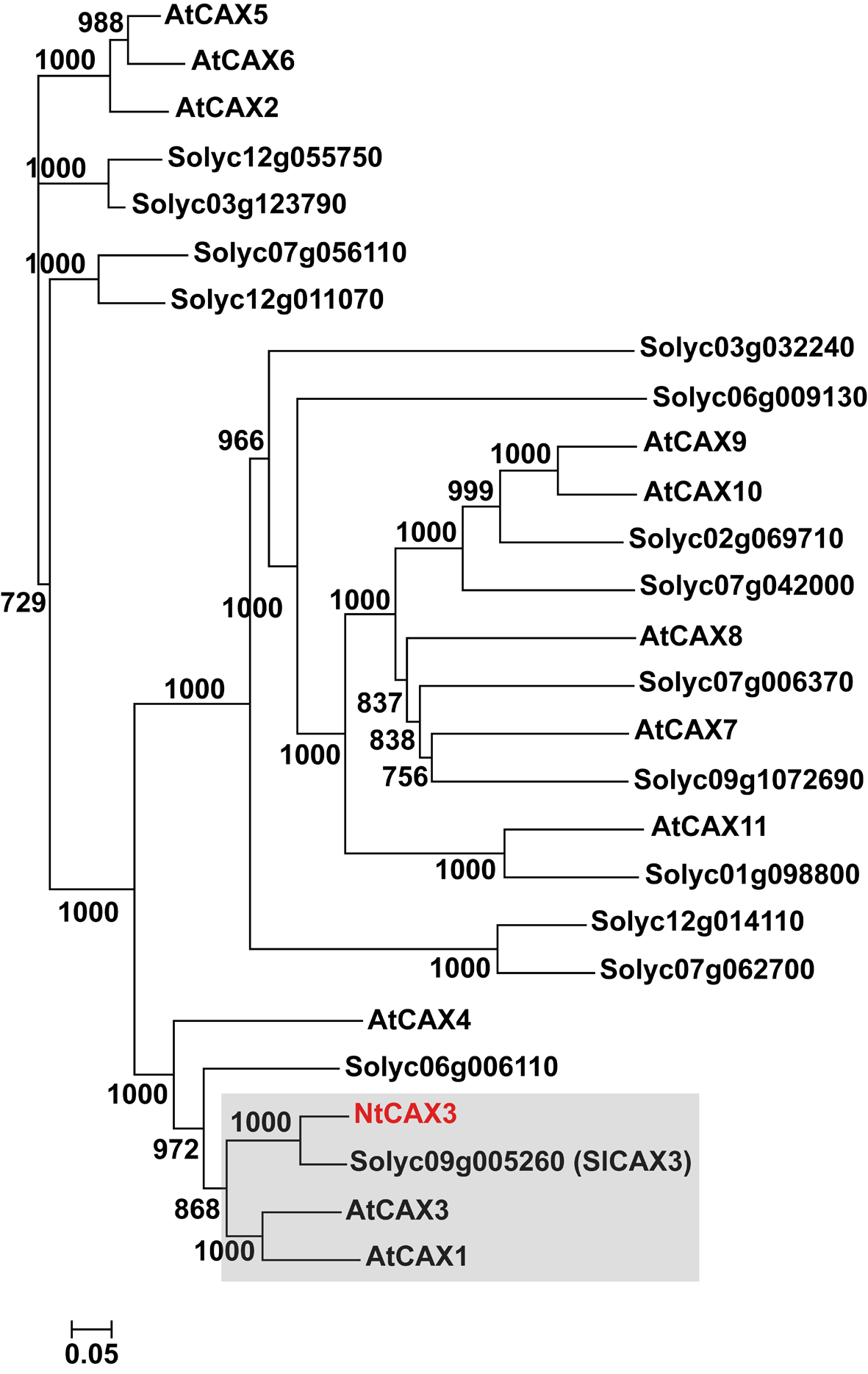

Supplement: Figure S5 — Phylogenetic analysis of NtCAX3 with CAXs from Arabidopsis and tomato. The phylogenetic tree was generated using ClustalX2.0 and MEGA4.0 softwares. AtCAXs are obtained from uniprot database (http://www.uniprot.org/). SlCAXs are obtained from plantGDB database (http://www.plantgdb.org/SlGDB/or ftp://ftp.plantgdb.org/download/Genomes/SlGDB/). Their accession numbers are as follows:AtCAX1(Q39253);AtCAX2(Q39254);AtCAX3(Q93Z81);AtCAX4(Q945S5);AtCAX5(Q8L783);AtCAX6(Q9LFZ8);AtCAX7(Q9FKP1);AtCAX8(Q9FKP2);AtCAX9(Q9LJI2);AtCAX10(Q9SYG9);AtCAX11(O04034);Solyc01g098800;Solyc02g069710; Solyc03g032240; Solyc03g123790; Solyc06g006110; Solyc06g009130; Solyc07g006370; Solyc07g042000; Solyc07g056110; Solyc07g062700; Solyc09g005260 (SlCAX3); Solyc09g072690; Solyc12g011070; Solyc12g014110; Solyc12g055750. (TIF) [file pone.0069881.s005.tif]
