# Supplementary material for: TaCIPK29, a CBL-Interacting Protein Kinase Gene from Wheat, Confers Salt Stress Tolerance in Transgenic Tobacco
Source: PLoS One. 2013 Jul 29;8(7):e69881. doi: 10.1371/journal.pone.0069881 (PMC3726728; doi:10.1371/journal.pone.0069881)
Supplement: Table S1 — Primers used for PCR analysis. (DOC) [file pone.0069881.s006.doc]

| Gene | Primer | Forward primer (5’-3’) | Reverse primer (5’-3’) |
| --- | --- | --- | --- |
| *TaCIPK29* | P1 | AAGCAGTGGTATCAACGCAGAGT | CCTTGAACTCCGTCTTGAACCAC |
| *TaCIPK29* | P2 | AAGCAGTGGTATCAACGCAGAGT | GATGAGGCTGGCGTCGTTGAA |
| *TaCIPK29* | P3 | GCGTGAAGCCTCAGGCAGTGT | AAGCAGTGGTATCAACGCAGAGT |
| *TaCIPK29* | P4 | CAGTGTGAAAAAGCTGCCGAGT | AAGCAGTGGTATCAACGCAGAGT |
| *TaCIPK29* | P5 | ACAGAATCCCAAGCCCCGAGAGA | GGTTTTCGTCCAGGTTCGTTGTA |
| *TaCIPK29* | P6 | ACGCGCAAGAAGGTCCACTT | ACACGAGCTGGCGGAAGTAA |
| *TaActin* | P7 | TGCTATCCTTCGTTTGGACCTT | AGCGGTTGTTGTGAGGGAGT |
| *TaCIPK29* | P8 | CGGAATTCATGCCGTCCGCCTCCAGCGC | CGGGATCCTCACACTGCCTGAGGCTTCACG |
| *TaCIPK29* | P9 | GCTCTAGAATGCCGTCCGCCTCCAGCGC | CGGGATCCTCACACTGCCTGAGGCTTCACG |
| *TaCIPK29* | P10 | TCAACGACGCCAGCCTCATCA | GTCCTCCTTGAACTCCGTCTTG |
| *TaCBL2* | P11 | TACCTCAAGGACATCACCACAAC | CAAAGGCTCCCAACAATACTAAA |
| *TaCBL3* | P12 | TAGTTCTCAGACATCCCTCATTAC | AACAAAGGCATTTCTGCTACG |
| *NtCBL2* | P13 | AAGCAGTGGTATCAACGCAGAGT | AAACCCAAGATTCCATTGTGCT |
| *NtCBL2* | P14 | TCTGCCTTATTGCCTCCTTCC | TCAAGCCCAAATCAGGTGTCC |
| *NtCBL2* | P15 | AAGAGGTGAAGCAAATGGTCGTT | TCAAGCCCAAATCAGGTGTCC |
| *NtCBL2* | P16 | CGGAATTCATGCTGCAGTGCTTAGGTTCT | CGGGATCCTCAGGTGTCCTCAACTCTTGAATGAA |
| *NtCBL3* | P17 | CGGAATTCATGTTGCAGTGCCTAGACGGTATC | CGGGATCCTCAGGTATCCTCAACTCTGGAATGA |
| *NtCBL3* | P18 | GGTGGTTCCTACCCTTGCTG | ATCTCAAATCAGGTATCCTCAACTC |
| *NtCAT1* | P19 | CGGAATTCATGGATCCATACAAGTACCGTCC | AACTGCAGTCATATGCTTGGTCTCACATTA |
| *NtPOX2* | P20 | CGGAATTCATGGCTTTTCGTTTGAGTCATTTG | CGGGATCCTCACATAGAAGCCACAGAGC |
| *NtCAT1* | P21 | AGGTACCGCTCATTCACACC | AAGCAAGCTTTTGACCCAGA |
| *NtPOX2* | P22 | CTTGGAACACGACGTTCCTT | TCGCTATCGCCATTCTTTCT |
| *NtSOS1* | P23 | CAAATGTTATCCCCCGAAAGC | CGGAGAACCTGAGGAAATGTGA |
| *NtNHX2* | P24 | ACTCATCCCCATTGGTCCG | AAGGAGTTCCACAAAAGCACGA |
| *NtNHX4* | P25 | CAAGAACTTCCGCACCCAC | GCAGTATCAAACGCAGAGGACC |
| *NtCAX3* | P26 | CGGTTTGGCAATAATTGTCACAG | CAACGATCATGCTTCAATCATCC |
| *NtACTIN* | P27 | TCCAGGACAAGGAGGGTAT | CATCAACAACAGGCAACCTAG |

**Table S1** Primers used for PCR analysis
