# Supplementary material for: TaCIPK29, a CBL-Interacting Protein Kinase Gene from Wheat, Confers Salt Stress Tolerance in Transgenic Tobacco
Source: PLoS One. 2013 Jul 29;8(7):e69881. doi: 10.1371/journal.pone.0069881 (PMC3726728; doi:10.1371/journal.pone.0069881)
Supplement: Table S2 — Sequence analysis of tobacco antiporters with the corresponding orthologs in Arabidopsis and tomato. (DOC) [file pone.0069881.s007.doc]

| Gene name | Orthologs | Query cover  (%) | E value | Max identity  /similarity (%) | GeneBank  Accession No. |
| --- | --- | --- | --- | --- | --- |
| NtSOS1 | SlSOS1 | 100 | 5e-166 | 89/93 | AJ717346 |
|  | AtSOS1/AtNHX7 | 98 | 2e-125 | 68/84 | NP_178307 |
| NtNHX2 | LeNHX2 | 98 | 2e-54 | 92/93 | CAC83608 |
|  | AtNHX5 | 83 | 2e-20 | 61/74 | AEE33089 |
| NtNHX4 | LeNHX4 | 100 | 5e-80 | 86/91 | AM261867 |
|  | AtNHX1 | 100 | 2e-55 | 65/74 | AAT95387 |
| NtCAX3 | SlCAX3 | 99 | 0.0 | 81/94 | AK3222020 |
|  | AtCAX3 | 96 | 0.0 | 71/83 | NP_190754 |
|  | AtCAX1 | 96 | 0.0 | 70/81 | CCH26476 |

**Table S2** Sequence analysis of tobacco antiporters with the corresponding orthologs in Arabidopsis and tomato
